# The gut-liver axis in hepatobiliary diseases

**DOI:** 10.1186/s41232-023-00315-0

**Published:** 2024-01-08

**Authors:** Masataka Ichikawa, Haruka Okada, Nobuhiro Nakamoto, Nobuhito Taniki, Po-Sung Chu, Takanori Kanai

**Affiliations:** Division of Gastroenterology and Hepatology, Department of Internal Medicine, Keio University School of Medicine, Shinanomachi, Tokyo, 1608582 Japan

**Keywords:** Intestinal bacteria, Gut-liver axis, Leaky gut, Primary sclerosing cholangitis, Fecal microbiota transplantation, Bacteriophage

## Abstract

Recent advances in the analysis of intestinal bacteria have led to reports of variations in intestinal bacterial levels among hepatobiliary diseases. The mechanisms behind the changes in intestinal bacteria in various hepatobiliary diseases include the abnormal composition of intestinal bacteria, weakening of the intestinal barrier, and bacterial translocation outside the intestinal tract, along with their metabolites, but many aspects remain unresolved. Further research employing clinical studies and animal models is expected to clarify the direct relationship between intestinal bacteria and hepatobiliary diseases and to validate the utility of intestinal bacteria as a diagnostic biomarker and potential therapeutic target. This review summarizes the involvement of the microbiota in the pathogenesis of hepatobiliary diseases via the gut-liver axis.

## Background

The human gastrointestinal tract harbors over 100 trillion intestinal bacteria from approximately 1000 species, and it is estimated that the total number of genes in these bacteria is estimated to be approximately 150 times greater than the number of human genes [1]. In the gut, intestinal bacteria establish an ecology by living off nutrients ingested by the host, and by interacting with the host and other bacterial species. Host physiology and disease pathogenesis can be influenced by intestinal bacteria through infection, short-chain fatty acids (SCFAs) and vitamin production, and bile acid metabolism [2]. Consequently, the host maintains its health while receiving both beneficial and detrimental influences from the gut microbiota. However, if the gut microbiota is disrupted by any factor, the host’s health is put at risk. In developed countries, for instance, allergic and autoimmune diseases are on the rise, which can be attributed to dysbiosis [3]. Other pathological conditions such as obesity [4], diabetes [5], colorectal cancer [6], and atherosclerosis [7] are also thought to be partly affected by intestinal microbiota. It is widely known that the composition of the intestinal microbiota differs greatly between patients with these diseases and healthy individuals.

The hepatobiliary system is in direct communication with the gastrointestinal tract via the portal vein and is continuously exposed to foreign substances (nutrients, enterobacteria-related substances, cytokines, etc.) of gastrointestinal origin. To deter harmful factors from entering the body, the liver acts as a biological firewall, maintaining a balance between immune response and immune tolerance. In recent years, groundbreaking advances in DNA sequencing of microbial genomes, in addition to transcriptome, proteome, and metabolome analysis, complemented by pathological studies using animal models, have dramatically improved our understanding of the composition and pathogenesis of the microbiome in a variety of diseases. In this review, the involvement of the microbiota in the pathogenesis of hepatobiliary diseases is outlined.

## Mechanisms of disease progression via intestinal bacteria and gut-liver axis

### The intestinal barrier and gut-vascular barrier

**Fig. 1:**
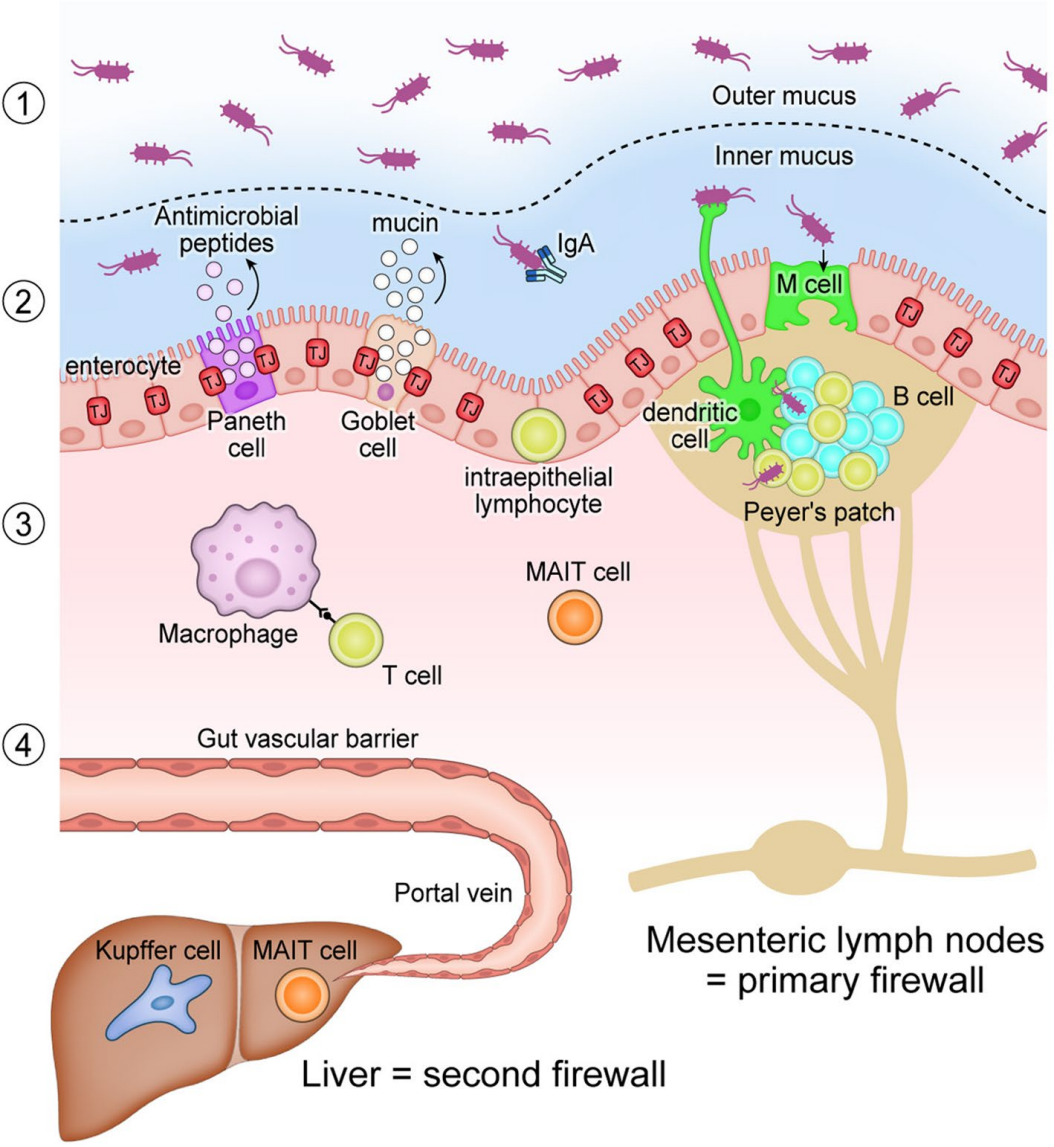
Major components of the intestinal barrier comprising the following four layers. (1) The mucus layer protects against microbial invasion by secreted substances such as mucin from goblet cells, IgA antibodies from the intestinal mucosal layer, and antimicrobial peptides from Paneth cells. This layer is divided into an outer mucus layer that supports the growth of commensal bacteria and an inner mucus layer that protects against bacterial invasion. (2) The epithelial layer contains enterocytes, goblet cells, Paneth cells, and M cells. Intraepithelial lymphocytes (IELs) are abundant. Tight junctions prevent physical bacterial invasion, while M cells sample luminal microorganisms for mucosal immune surveillance. (3) The mucosal intrinsic layer possesses an active immune barrier that contains T cells, B cells, macrophage, dendritic cells (DCs), and innate immune-like cells such as MAIT cells. DCs extend their projections to directly inspect and transport microbes to mesenteric lymph nodes (MLNs). (4) The gut-vascular barrier (GVB), consisting of a structured endothelium, is an independent barrier that regulates the translocation of luminal bacteria and their ligands as well as innocuous food antigens. MLNs act as primary firewall that prevents microbes from entering the systemic circulation. When bacteria invade the GVB, they reach the liver via the portal vein. The liver, which is enriched with immune cells such as Kupffer cells and MAIT cells, serves as second firewall. IgA, immunoglobulin A; M cells, microfold cells; MAIT cells, mucosal-associated invariant T cells; TJ, tight junction

Estimates suggest that humans have more than 100 trillion intestinal bacteria in their bodies, and the gastrointestinal tract is constantly exposed to these microorganisms [1]. The intestinal barrier has four layers to protect against bacterial attack: the mucus layer facing the lumen, the epithelial layer that prevents physical invasion through tight junctions, the mucosal intrinsic layer that possesses an active immune barrier, and the gut-vascular barrier (Fig. 1). The mucus layer, the primary barrier, protects against microbial adhesion and invasion by secreted substances such as mucin, immunoglobulin A (IgA), and antimicrobial peptides (AMPs). The intestinal epithelium, on the other hand, is divided into two layers: an outer mucus layer, which is coarse and supports the growth of commensal bacteria, and an inner mucus layer, which is dense, sterile, contains antibacterial peptides, and protects against bacterial invasion. Goblet cells continually produce mucin in this layer, while IgA antibodies secreted from the intestinal mucosal layer into the intestinal lumen effectively bind and form complexes with bacteria, stimulate intestinal mucus secretion, prevent bacterial attachment to the intestinal mucosa, and neutralize toxins produced by bacteria. AMPs found in this layer include defensins, cathelicidines, resistin-like molecules, bactericidal and permeability-inducing proteins, and lectins [8]. Mature defensins possess antimicrobial activity that disrupt microbial membranes. Lectins are known to bind to cell wall peptidoglycans of gram-positive bacteria and have a bactericidal function [9, 10]. The composition of these mucus barriers is also defined by their microflora [11], which not only serves as the first line of defense but also acts as a source of nutrients and a niche for colonization, enabling the microflora to survive peristaltic action.

The second barrier, the epithelial layer, contains enterocytes, goblet cells, enteroendocrine cells, Paneth cells, and microfold cells (M cells) [12]. These cells cooperate together to protect the intestine from damage caused by the microflora. This barrier has multiple functions, including a physical barrier, formed by adjacent epithelial cells sealed by tight junctions. It also acts as an electrical barrier, in which the positive charge of defensins attracts the negative charge of the microbiota, resulting in efficient antimicrobial activity. Additionally, it functions as a chemical barrier, releasing a range of AMPs from epithelial cells. Moreover, a series of mucosal immune cells, described below, patrol the epithelium. There are specialized lymphocytes called intraepithelial lymphocytes (IELs), which are primarily *cluster of differentiation* (CD)8-positive T cells that possess cytotoxic activity and prevent the spread of infection by inducing the apoptosis of microbially infected epithelial cells. On the other hand, M cells are specialized intestinal epithelial cells that sample luminal microorganisms for mucosal immune surveillance.

The third barrier, the mucosal intrinsic layer, contains lymphocytes, mainly CD4+ T cells, IgA-producing plasma cells, and innate immune-like cells such as invariant natural killer T (NKT) cells and mucosal-associated invariant T (MAIT) cells. NKT cells recognize lipids presented on CD1 molecules [13], while MAIT cells recognize riboflavin metabolites presented on MR1 molecules [14]. IL17-producing CD4+ T (Th17) cells release interleukin (IL)-17A, IL-17F, and IL-22, which enhance tight junction molecules between epithelial cells and promote epithelial cell regeneration. A switch to a Th1 pattern of tumor necrosis factor alpha (TNF-a) and interferon-gamma (IFN-γ) production and a concomitant depletion of Th17 in the native layer has been reported in a rat model of liver cirrhosis as cirrhosis progresses into the decompensated phase [15]. Gut-associated lymphoid tissue (GALT) is the largest immune organ in the human body. The first line of defense, the innate immune system, detects pathogenic patterns by recognizing the pattern recognition receptor (PRR) on intestinal epithelial cells. Dendritic cells (DCs) located in the sub-epithelium open tight junctions between epithelial cells and extend their projections to directly inspect and transport microbes to mesenteric lymph nodes (MLNs). MLNs are regarded as the primary firewall of the intestinal lymphatic circulation [16], and indigenous bacteria transported to MLNs in a healthy state are prompted to undergo apoptosis by a local immune response [17]. In response to bacterial translocation (BT), intestinal epithelial cells release chemokines and induce mobilization of DCs to the mucosa. Activated and mature intestinal DCs induce B and T cells to elicit acquired immunity. Microbial antigens presented to B cells induce IgA responses specific for commensal bacteria and protect against enterobacterial invasion into the deep intestinal tract and beyond. Of the aforementioned immune systems centered on GALT, TNF is of particular interest because mouse experiments have demonstrated that TNF disrupts tight junctions in the epithelium [18]. Similarly, TNF secretion is increased in the MLN and serum of patients with cirrhosis has been reported to predict post-transplant bacterial infection [19]. In summary, increased TNF concentrations in GALT are crucial in the development of pathological BT in cirrhosis. Additionally, genetic polymorphisms in toll-like receptor 2 (TLR2) proteins, which are expressed on immune cells and are able to recognize pathogen-associated molecular patterns (PAMPs) from gram-positive microorganisms, can increase the risk of spontaneous bacterial peritonitis (SBP) [20]. TLR2-deficient mice have significantly reduced BT, supporting a facilitative role for TLR2 in pathological BT [18].

Finally, a fourth barrier, the gut vascular barrier, has recently been discovered beneath the intestinal epithelium [21]. Composed of endothelial cells coupled with pericytes and enteric glial cells, this barrier has similarities to the blood-brain barrier and is regulated by the Wnt/β-catenin signaling pathway [22]. It has become recently evident that BT is caused by gut-vascular barrier (GVB) dysfunction in the early stages of various liver-related pathologies, including non-alcoholic steatohepatitis (NASH) induced by a high-fat diet [23], alcoholic hepatitis [24], and liver metastases of colorectal cancer [25]. Interestingly, obeticholic acid was shown to restore reduced ileal farnesoid X receptor (FXR) signaling, improve mucus-producing function, and stabilize GVB in cirrhotic rats [26], suggesting that the nuclear receptor, FXR, partially regulates mucus production and GVB in cirrhosis.

Despite the various protective barriers described above, bacteria can still circumvent them and pose a risk of systemic infection. The majority are filtered out by MLNs, which prevent microorganisms from entering the systemic circulation. Some bacteria, however, breach the underlying GVB, enter the portal circulation, and reach the liver, considered as the second firewall [16]. In the liver, Kupffer cells remove the bacteria from blood vessels [26]. MAIT cells are also known to play a protective role in bacterial infections and are particularly abundant in the human liver, accounting for 50% of all T cells [27]. It is suggested that hepatic MAIT cells are highly activated in the liver and likely play a protective role against various extracellular and intracellular bacteria, fungi, and viruses as part of the liver firewall through the abundant and rapid production of IFN-γ and IL-17 [28], but to date, there are limited data to demonstrate this phenomenon and further investigation is needed.

Responses to acute inflammation can restore barrier failure, but persistent barrier failure can lead to uncontrolled immune responses in the gut microenvironment. Chronic inflammation downregulates tight junctions and impairs intercellular junctions, resulting in a leaky gut. Furthermore, inflammation affects the regulation of the mucosal immune system and contributes to the development of intestinal and mesenteric diseases, the pathogenicity of which involve the immune system [29]. In fact, gut permeability has been reported to be involved in the pathogenesis of a host of diseases, including intestinal diseases such as celiac disease [30] and inflammatory bowel disease (IBD) [31], as well as extraintestinal diseases like rheumatoid arthritis [32], multiple sclerosis [33], diabetes mellitus [34], and obesity [35]. Similarly, in the pathogenesis of liver cirrhosis, increased gut permeability has been demonstrated in both humans and animal models, especially in the presence of ascites [36–38]. As cirrhosis progresses, these barriers are disrupted at each stage of the disease, eliciting hepatobiliary damage via excessive immune responses in the liver to intestinal bacteria and their metabolites, in addition to direct toxicity. While the diversity of the intestinal microbiota is reduced in cirrhosis, as discussed below, it is more likely to result in small intestinal bacterial overgrowth (SIBO). Indeed, SIBO is one of the main factors promoting BT in cirrhosis, and the occurrence of BT in MLNs in experimental models is associated with SIBO [36]. There are no clinical markers to strictly monitor BT, but the surrogate markers of pathological BT, such as systemic endotoxin levels, increase progressively in relation to the severity of cirrhosis by the Child classification [39, 40].

### Immune cells and their interaction with intestinal bacteria

The innate immune system is closely associated with commensal bacteria. AMPs, with their antimicrobial activity, are produced primarily by Paneth cells, which also contribute to microbiome organization. PRRs, especially TLRs, are innate immune sensors that respond to microbial signals by recruiting specific adaptor elements, including myeloid differentiation primary response 88 (MyD88), and activating transcription factors, such as nuclear factor kappa B (NF-κB) [41]. TLRs are also abundantly expressed in various cells in the liver, including Kupffer cells, dendritic cells, hepatic stellate cells, endothelial cells, and hepatocytes. Animal studies have shown that hepatic TLR4 signaling induced by a lipopolysaccharide, a bacterial cell wall component, can cause hepatitis and fibrosis [42]. Other PRRs known to shape the composition of the gut microbiota include NOD-like receptors (NLRs) and nucleotide-binding oligomerization domain-containing protein 1 (NOD1), which functions as an endogenous sensor to maintain gut homeostasis [43]. In addition to components of microbial origin, the NLR family recognizes damage-associated molecular patterns (DAMPs) released from injured cells. Activated NLRs associate with pro-caspase-1 via adapter proteins to form large complexes called inflammasomes. Recent studies have reported that inflammasome signaling within hepatocytes, macrophages, and Kupffer cells is associated with the development of inflammatory liver injury [44]. Innate immune cells are most abundant in the liver, which is constantly being exposed to indigenous bacteria through the portal vein. These immune cells include Kupffer cells, which account for 80–90% of all indigenous macrophages in the body, as well as monocyte-derived macrophages, natural killer cells, natural killer T cells, γδ T cells, MAIT cells, and lymphoid cells. Myeloid and lymphoid resident immune cells are abundant in the portal region of the hepatic lobule, and Myd88-dependent signaling of hepatic sinusoidal endothelial cells induced by commensal bacteria contributes to asymmetric zonation in the liver lobule [45].

Gut bacteria and the acquired immune system have been shown to interact. Studies using germ free mice have reported that gut bacteria which ferment dietary fiber into SCFAs are essential for the differentiation of regulatory CD4+ T cells (Treg) in the colon [46]. A proportion of the primary bile acids secreted into the intestine escape the enterohepatic circulation and enter the colon, where intestinal bacteria convert them into biologically active secondary bile acids. Secondary bile acids have also been reported to be involved in the colonic regulation of forkhead box P3 (FOXP3)-positive Treg cells differentiation [47]. Th17 cells possess a protective aspect that defends against infection and an inflammatory immune response that contributes to the development of autoimmune and other diseases. Th17 cells induced by segmented filamentous bacteria in mice are non-inflammatory, whereas Th17 cells stimulated by *Citrobacter rodentium* are a source of inflammatory cytokines [45]. Recent studies have shown that gut bacteria promote the long-term survival of activated CD8+ T cells via metabolites [48]. Some bile acid metabolites directly affect the differentiation of acquired immunity. 3-oxoLCA, a derivative of lithocholic acid (LCA), inhibits Th17 cell differentiation. Conversely, isoalloLCA promotes the differentiation of Treg cells. In mice, treatment with 3-oxoLCA and isoalloLCA reduced Th17 and increased Treg cell differentiation in the intestinal lamina propria [45]. Thus, it is suggested that intestinal bacteria, along with their metabolites and bile acids, directly influence the acquired immune system and directly contribute to the pathogenesis of hepatobiliary diseases.

### Bile acids and the enterohepatic circulation

**Fig. 2:**
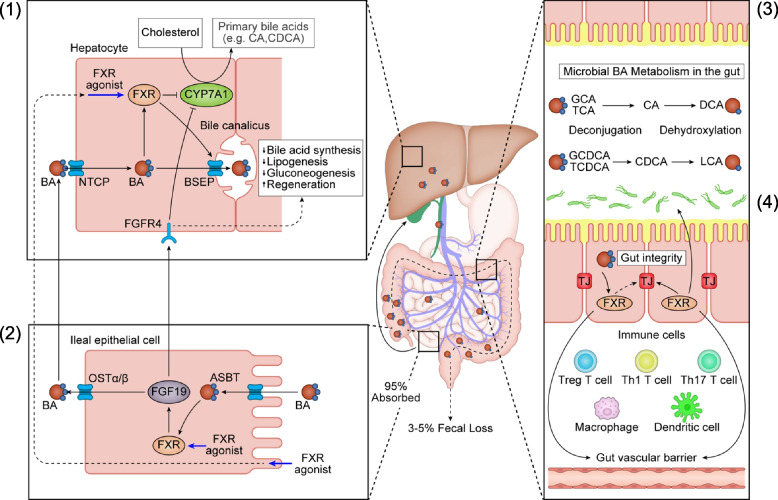
Bile acid (BA) biosynthesis and circulation along the gut-liver axis. (1) Hepatocellular BA homeostasis. The primary BAs such as cholic acid (CA) and chenodeoxycholic acid (CDCA) are synthesized from cholesterol via BA synthesis enzymes such as CYP7A1. The hepatic uptake of BAs is mediated via sodium/taurocholate cotransporting polypeptide (NTCP). BAs, either taken up from portal blood or newly synthesized, are excreted into bile canaliculi via the bile salt export pump (BSEP). BAs inhibit CYP7A1 and induce BSEP via activation of FXR and NTCP transcription. Thus, the load of BA is maintained. BA synthesis is also inhibited by the FXR agonist and fibroblast growth factor receptor 4 (FGFR4), which is bound to intestinal FGF19. FGFR4 also inhibits lipogenesis and gluconeogenesis while promoting regeneration. (2) Intestinal BA transport in the ileal epithelial cells. In the terminal ileum, 95% of BAs are reabsorbed into ileal epithelial cells via the apical sodium-dependent bile acid transporter (ASBT). A basolateral organic solute transporter alpha and beta (OSTα/β) heterodimer mediates the efflux of BA from ileal epithelial cells into the portal blood for circulation back to the liver. In the ileum, BAs and FXR agonist activate FXR and induce FGF19, which circulates to the liver and binds to FGFR4. Thus, the enterohepatic circulation associated with BAs is formed. (3) Microbial BA metabolism in the gut. In the lumen of the distal ileum and colon, gut microbial bile salt hydrolase (BSH) deconjugates glycine and taurine conjugated BAs, and microbial 7α-dehydroxylase removes the 7α-hydroxyl group to covert the primary BAs, CA and CDCA, to the secondary bile acids, DCA and LCA. (4) Role of BAs in gut integrity. BAs affect intestinal microbiota and effect epithelial barrier integrity via FXR stimulation. The activated FXR regulates the tight junction (TJ), mucin production, and gut-vascular barrier. BAs also have anti-inflammatory and immunomodulatory effects on both innate and adaptive immune cells, preventing inflammatory reactions that would damage intestinal integrity. ASBT, apical sodium-dependent bile acid transporter; BA, bile acid; BSEP, bile salt export pump; BSH, bile salt hydrolase; CA, cholic acid; CDCA, chenodeoxycholic acid; CYP7A1, cholesterol-7α-hydroxylase; DCA, deoxycholic acid; FGF19, fibroblast growth factor 19; FGFR4, fibroblast growth factor receptor 4; FXR, farnesoid X receptor; LCA, lithocholic acid; NTCP, sodium/taurocholate cotransporting polypeptide; OSTα/β, organic solute transporter alpha and beta; TJ, tight junction

In addition to lipid digestion and absorption, bile acids send important signals that regulate hepatic metabolism, gut microbiota composition, and intestinal permeability. Through bile acid signaling, nuclear receptors such as FXR and G protein-coupled receptors (GPCRs) like Takeda-G-protein-receptor-5 (TGR5) regulate bile acid balance, lipid and sugar homeostasis, innate and acquired immunity [49–51]. Bile acid transport and signaling are summarized in Fig. 2.

Bile acids are synthesized from cholesterol in the liver, and after conjugation with taurine and glycine, they are excreted into bile and partly metabolized by intestinal bacteria into secondary bile acids. The majority of primary and secondary bile acids go through the enterohepatic circulation. They are reabsorbed in the small and large intestine and then returned to the liver through the portal blood [52–54]. The secretion and reabsorption of bile acids are efficiently mediated by bile acid transporters, and only 3%–5% are eliminated in the feces [50, 54]. Primary bile acids, such as cholic acid and chenodeoxycholic acid, are converted to secondary bile acids, including deoxycholic acid and lithocholic acid, through dehydration and dehydroxylation via intestinal bacteria. When bile acids are reintroduced into the liver, they are re-harbored and re-hydroxylated before being excreted into bile. Thus, a close relationship between intestinal bacteria and bile acid metabolism exists, and species such as *Firmicutes*, *Bacteroides*, *Eubacterium*, and *Clostridium* convert bile acids through hydrolysis. Bacterial genotypes are also involved in the dehydroxylation, oxidation, and epimerization required for bile conversion to secondary bile acids [50, 55–57]. The secondary bile acids, deoxycholic acid and lithocholic acid, are 7-dehydroxylated bile acids of cholic acid and chenodeoxycholic acid, respectively. These undergo epimerization by enteric bacteria to produce iso-bile and allo-bile acids and are subjected to oxidation to produce oxo-bile acids. It is also known that certain bile acids activate host bile acid-related receptors such as FXR and TGR5 [50].

Bile acids directly contribute to the amount and composition of intestinal bacteria. In the jejunum, bile acids act directly on bacteria mainly through cell membrane toxicity and inhibition of bacterial metabolism [58]. In the ileum, antimicrobial activity is mainly achieved through FXR-mediated bile acid signaling [59–61]. In contrast, some bacteria use bile acids, such as taurine-conjugated bile acids, as an energy source for growth [57]. For instance, mice that were fed a dairy fat diet showed increased levels of taurocholic acid, resulting in an increase in *Bilophila wadsworthia*, a bacterium associated with IBD [62]. Furthermore, exposure to bile acids in the small intestine has been found to enhance resistance to enterohemorrhagic *Escherichia coli* [63] and to alter the toxicity of *Clostridioides difficile* [64]. Moreover, bile acids are known to be involved in the improvement of the intestinal barrier. In fact, the involvement of bile acids in each layer of the intestinal barrier, such as maintenance of the mucosal layer [65], tight junctions [61, 66], and GVB [26], has been reported. In a mouse model of bile duct injury, administration of an FXR agonist has been reported to stabilize intestinal barrier function and improve portal pressure [67, 68]. As mentioned above, certain bile acids exert anti-inflammatory effects by regulating innate and acquired immunity via FXR and TGR5 [50, 66].

Bile acid-related therapeutics targeting FXR and its downstream, fibroblast growth factor (FGF) 19, are emerging as crucial therapeutic options for cholestasis and metabolism-related liver diseases [50, 69]. Ursodeoxycholic acid (UDCA), a hydrophilic bile acid, is the first enterohepatic circulating agent that promotes bile secretion and inhibits cholestasis [70]. Similarly, steroidal and non-steroidal FXR ligands exert different pharmacological effects. Steroidal FXR ligands, such as obeticholic acid, act via the enterohepatic circulation [71], whereas nonsteroidal FXR ligands are confined to the intestinal tract [72].

### Metabolites (SCFAs, amino acids, AhR, etc.)

Normal carbohydrates, proteins, and fats are digested in the small intestine, but non-digestible carbohydrates like dietary fiber are produced as SCFAs, including acetic acid, propionic acid, and butyric acid in the large intestine through intestinal bacteria fermentation and are involved in promoting mammalian health [73]. These SCFAs play a role in energy metabolism and immunity through GPCRs, such as GPR41 and GPR43 [74, 75]. Butyric acid serves as an energy source for colonic cells, acetic acid is involved in lipid and cholesterol biosynthesis in the liver, and propionic acid contributes to glycogenesis [75–77]. SCFAs also contribute to improving intestinal barrier function by strengthening the mucin layer and facilitating the release of antimicrobial peptides [75]. In addition, SCFAs enhance Treg induction and suppress intestinal inflammation, thereby maintaining intestinal homeostasis [78]. In the liver, it has been reported that feeding SCFAs to NASH model mice on a choline- and methionine-deficient diet reduces hepatic steatosis and inflammation [79] and that feeding SCFAs to HBV-encoded oncogene X protein (HBx) transgenic hepatocellular carcinoma (HCC)-bearing mice slows the progression of HCC [80].

Tryptophan, an essential amino acid, is metabolized and converted to indole by intestinal bacteria, which acts on aryl hydrocarbon receptors (AhRs) expressed in the intestinal epithelium, which in turn contributes to the survival and differentiation of the intestinal epithelium [81]. In studies using an enteritis model, a decrease in indole-3-acetic acid, an AhR agonist, induces intestinal inflammation via decreased IL-22 production, suggesting that AhR has anti-inflammatory properties [82]. Furthermore, this anti-inflammatory action is not only limited to the intestinal tract but also extends to the whole body, including the liver [83]. AhR agonists are known to decrease in cases of metabolic syndrome and fatty liver and administering AhR agonists to mice models has been found to reduce liver injury [84]. Additionally, research has found that ethanol-induced liver injury is exacerbated in mice lacking intestinal epithelial-specific AhR compared to the control group, indicating that AhR is involved in liver pathology through intestinal-hepatic interphase [85]. Although, AhR is also expressed in the constituent cells of the liver [86], the detailed mechanism of its involvement in liver pathology awaits further elucidation.

## Microbiota and hepatobiliary diseases

### Primary biliary cholangitis

Primary biliary cholangitis (PBC) is an autoimmune biliary disease that affects the bile ducts of the liver and involves the destruction of relatively small intrahepatic ducts by an autoimmune mechanism, leading to chronic bile stasis and liver cirrhosis [87]. Clinically, PBC is characterized by elevated serum alkaline phosphatase (ALP), anti-mitochondrial antibodies (AMA), and high IgM levels. Additionally, it has been reported that intestinal bacteria, particularly vaginal and urinary tract infection-causing bacteria, are involved in the pathogenesis of PBC [88, 89]. The major AMA antigen, pyruvate dehydratase complex E2 component (PDC-E2), is conserved across species and cross-reacts with microbial antigens such as *Escherichia coli* (*E. coli*) and *Novosphingobium aromaticivorans*. Autoreactive T cells from PBC patients, which implies that molecular autoimmunity is due to molecular homology, has been postulated as a possible mechanism of pathogenesis [90, 91]. In fact, the induction of PBC-like cholangiopathy in mice transplanted with *Novosphingobium aromaticivorans* suggests the involvement of specific intestinal bacteria in the pathogenesis of the disease [91].

UDCA is widely used as a treatment for PBC and has been shown to improve hepatobiliary enzymes and prognosis. It has been reported that *Ruminococcus* spp., which is capable of producing UDCA, are decreased in the stools of PBC patients [92]. Their absence may directly contribute to the pathogenesis of PBC through the disruption of bile acid production [92]. Recently, an analysis of the intestinal bacteria in the stools of PBC patients before and after UCDA treatment revealed that *Haemophiles*, *Veillonella*, *Clostridium*, *Lactobacillus*, *Streptococcus*, *Pseudomonas*, and *Klebsiella* species were increased in untreated PBC patients and that 6 months of UDCA treatment partially restored the dysbiosis [93]. Functional analysis of *Enterobacteriaceae* and *Klebsiella spp*. exposed a positive correlation with their ability to penetrate the intestinal epithelium, indicating that BT through the colonic epithelium may be a common pathological mechanism utilized by certain intestinal bacteria in biliary congestion diseases similar to PSC, which will be discussed later.

### Primary sclerosing cholangitis

Primary sclerosing cholangitis (PSC) is an autoimmune hepatobiliary disease characterized by multiple and diffuse stenosis of relatively large bile ducts accompanied by biliary stasis [94]. While there have been reports of the improvement of hepatobiliary enzymes with UCDA and bezafibrate use, the long-term prognostic value of these drugs remains unclear; thus, liver transplantation is the only curative treatment [95]. In addition, immunosuppressive therapies such as steroids and anti-TNFa drugs have shown little clinical benefit in PSC [96].

PSC is clinically characterized by a high complication rate of IBD (60–80% in Europe and the USA, 30–50% in Asia) [97], implying that an enterohepatic correlation is involved in its pathogenesis. Analysis of the gut microbiota in a large cohort of PSC patients in Europe showed that the gut microbiota in the stools of PSC patients was significantly less diverse than that of healthy controls [98] with an increase in *Enterococcus*, *Streptococcus*, *Lactobacillus*, *Fusobacterium*, *Veillonella*, etc. [98, 99]. Furthermore, among these enterobacteria, *Enterococcus* has been reported to be strongly correlated with serum ALP levels, while *Fusobacterium* and *Veillonella* have been associated with intestinal inflammation as assessed by fecal calprotectin [100]. In particular, *Veillonella* was reported to be positively correlated with the PSC Mayo risk score in a Norwegian study [101] and was detected at a higher rate in PSC patients with cirrhosis in a Belgian study [99], implying an association with clinical characteristics. Moreover, prospective clinical trials conducted overseas that target intestinal bacteria through oral antimicrobial therapy have shown significant reductions in serum hepatobiliary enzymes (Table 1), indicating a potential association between PSC and intestinal bacteria [102–104].

**Table 1:** Clinical trials targeting the microbiota in the treatment for PSC

Treatment	Drug	Study design	n	Duration	Outcome	Reference
Antibiotic	Metronidazole (< 75 kg 600 mg, > 75 kg 800 mg) or placebo	RCT	80	3 years	ALP -52% (metronidazole) vs -38% (placebo)	Farkkila et al. 2004 [102]
Antibiotic	Vancomycin (125 mg or 250 mg qid) or metronidazole (250 mg or 500 mg tid)	RCT	35	12 weeks	ALP -40% and -46% (vancomycin 125 and 250 mg) vs +13% and -33% (metronidazole 250 and 500 mg)	Tabibian et al. 2013 [103]
Antibiotic	Vancomycin (125 mg qid) or placebo	RCT	29	12 weeks	ALP -53% (vancomycin) vs -8% (placebo)	Rahimpour et al. 2016 [104]
Probiotic	4 lactobacilli, 2 *Bifidobacterium*	RCT, cross over	14	12 weeks × 2 plus wash-out	No change in liver biochemistry	Vleggaar et al. 2008 [105]
FMT	Faecal sample from a healthy donoradministered during colonoscopy	Open-label pilot study	10	24 weeks observation	No change in liver biochemistry Gut microbiota profiles were changed with increased diversity, persisting for up to 24 weeks	Allegretti et al. 2019 [106]

*FMT* Fecal microbiota transplantation, *RCT* Randomized clinical trial

As the link between PSC and intestinal bacteria has been explored using clinical samples as described above, further investigations are underway to elucidate the pathogenesis of PSC via the gut-liver axis. In a study of the pathogenesis of bile duct injury, dextran sodium sulfate (DSS) administration induced bile duct injury but was attenuated by antimicrobial administration, indicating the involvement of intestinal barrier function and intestinal bacteria [107]. The fact that spontaneous bile duct injury in NOD.c3c4 mice was alleviated when they were sterile strongly suggests the involvement of intestinal bacterial [108]. On the other hand, in multidrug-resistant 2 deficient (Mdr2-/-) mice with spontaneous cholangiopathy, sterilization and the use of broad-spectrum antibiotics exacerbated cholangiopathy [109, 110]. In a sterile environment, secondary bile acids are not produced and the FXR antagonist β-muricholic acid accumulates, which may inhibit the FXR/FGF15 pathway that suppresses bile acid synthesis, resulting in the excessive production of bile acids [111]. It has also been documented that an abundance of *Lactobacillus gasseri* in the intestinal microbiota of Mdr2-/- mice [112] reduced the intestinal barrier function and allowed the bacterium to migrate into the liver, causing an increase in IL-17-producing T cells and NOD-like receptor protein 3 (NLRP3) inflammasomes in the liver [113]. More interestingly, feces from Mdr2-/- mice exhibited decreased diversity of intestinal microbiota and transfer of these feces to wild-type mice induced inflammasome-associated liver injury [113]. These results suggest that the intestinal microbiota itself, when altered by bile acids, may also be involved in the induction of biliary disease.

**Fig. 3:**
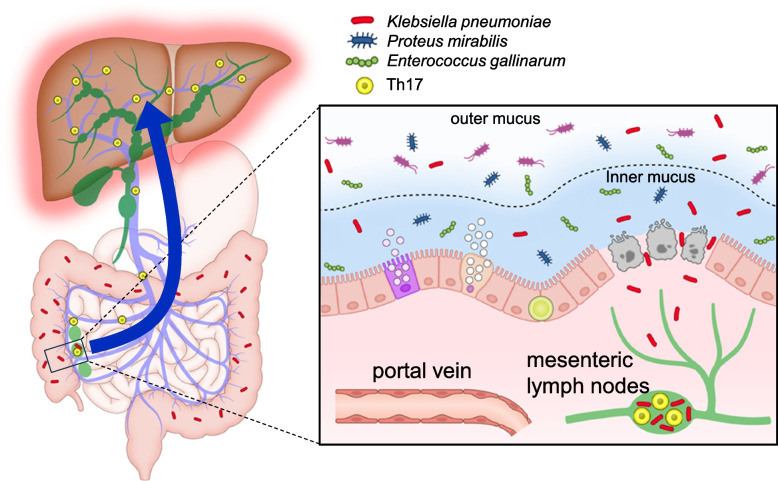
Role of the role of pathobionts in intestinal barrier dysfunction and liver inflammation via he Th17 immune response in patients with PSC. *Klebsiella pneumoniae*, *Proteus mirabilis*, and *Enterococcus gallinarum* are prevalent in patients with PSC. Of these pathobionts, *Klebsiella pneumoniae* damages the colonic epithelium and translocates to the mesenteric lymph nodes, where it induces Th17 with other pathobionts, contributing to the exacerbation of hepatobiliary inflammation. Other pathobionts such as *Enterococcus faecalis* and *Escherichia coli*, along with their metabolites may contribute to the pathogenesis of PSC by directly entering the portal circulation

Despite suggestions that intestinal bacteria and the gut-liver axis may be implicated in bile duct injury in mouse models of PSC and cholangiopathy, the pathogens directly driving the pathogenesis of PSC and their detailed mechanisms remain unclear. To elucidate the pathogenesis of PSC, we will review the most recent studies that have been reported. These studies have shown that treating Mdr2-/- mice with vancomycin exacerbated bile duct injury and caused a decrease in SCFA-producing *Lachnospiraceae* and in SCFAs themselves, while *Enterococcus faecalis* (*E. faecalis*), a member of the *Lachnospiraceae* family, was unaffected by vancomycin treatment. Transfer of *E. faecalis* and *E. coli* to Mdr2-/- mice resulted in the exacerbation of bile duct injury and increased lethality, demonstrating that they are directly involved in bile duct injury. In contrast, the transfer of *Lachnospiraceae* and SCFAs reduced bile duct injury in mice. The Mayo risk score of patients with PSC showed a positive correlation with *E. faecalis* and *E. coli* and a negative correlation with *E. faecalis* and *Lachnospiraceae*, indicating that *E. faecalis* and *E. coli* may be directly involved in the pathogenesis of PSC [114]. In a study conducted on humanized microbiota mice model in which stools from PSC patients with IBD were inoculated to germ-free mice, Th17 was induced in the liver, and bile duct injury was exacerbated when these mice were exposed to 3,5-diethoxycarbonyl-1,4-dihydrocollidine (DDC). These findings suggest the presence of intestinal bacteria in the stools of PSC patients that induce immune responses in the liver and aggravate bile duct injury. When each organ of the mice was cultured in a sterile manner, no intestinal bacteria were isolated from the liver or spleen, while *Klebsiella pneumoniae* (*Kp*), *Proteus mirabilis* (*Pm*), and *Enterococcus gallinarum* (*Eg*) were detected in the MLNs. The inoculation of these three bacteria to germ-free mice led to the induction of immune responses in the liver and exacerbation of bile duct damage, implying that these bacteria are directly involved in the pathogenesis of the disease via bacterial translation (Fig. 3). The induction of Th17 in the liver was partially canceled by the use of antibiotics sensitive to the bacteria. In particular, *Kp* was involved in the disruption of barrier function and migration to lymph nodes by perforating the colonic epithelium [115]. These three bacteria were detected at high rates in the stools of patients with PSC. Notably, serum ALP levels were higher and transplant-free survival tended to worsen in patients carrying *Kp* and *Eg* than in non-carriers [116]. Consistently, the Mayo PSC risk score, Fibrosis-4 score, and transplant-free survival were significantly worse in patients carting *Kp* in the recent Norway cohort [117], implying that this organism has the potential to be utilized as a biomarker and therapeutic target.

### Biliary tract cancer

The liver and bile ducts, which are sterile under steady state conditions, can be exposed to the gut microbiota through the gut-liver axis when the gut barrier is disrupted, leading to the development of liver disease, particularly bile duct disease. The biliary tract has an innate immune system that recognizes PAMPs by means of TLRs. When enteric bacteria invade the biliary tract, TLRs bind to bacterial cell wall components, including lipopolysaccharides (LPS), causing bile duct cells to release inflammatory cytokines for pathogen elimination [118, 119]. Chronic activation of TLRs and the subsequent chronic inflammation of bile duct cells are associated with cholangiocyte proliferation and neoplastic transformation, which may lead to the development of various biliary tract diseases [120]. Indeed, activation of TLR4 and high expression of the TLR4 gene are associated with cholangiocarcinoma (CCA) progression and worse disease outcomes, while lower TLR4 levels are associated with tumor growth suppression [121, 122].

The role of the intestinal microbiota in cancer pathology has been extensively studied in recent years, and both its antitumor and tumor-promoting effects have been observed. In support of the tumor-promoting effects of intestinal bacteria in CCA, a study using a mouse model showed that disruption of the intestinal barrier leads to accumulation of bacteria and LPS in the liver and biliary tract via the portal circulation, resulting in the mobilization of immunosuppressive cells to these areas [123]. Furthermore, Zhang et al. demonstrated marked changes in the gut microbiota and the detection of high levels of bacterial RNA in the portal vein in mice with PSC-like lesions after bile duct ligation. In this study, gram-negative bacteria/LPS invading the liver through the portal vein induced the accumulation of CXC chemokine receptor (CXCR2+) polymorphonuclear myeloid-derived suppressor cells via CXC motif chemokine ligand 1 (CXCL1) production from hepatocytes and promoted cholangiocarcinoma growth [124]. However, studies on tumor-associated enterobacteria in patients with intrahepatic CCA are limited and may reflect the difficulty in accessing bile samples compared to stool samples.

Dangtakot et al. performed a comparative study of the microbiota of bile fluid from 30 patients with intrahepatic cholangiocarcinoma and choledocholithiasis. Bacteria of the genera *Enterobacter*, *Pseudomonas*, and *Stenotrophomonas* were significantly more frequent in bile samples from patients with intrahepatic cholangiocarcinoma compared to those with cholelithiasis [125]. Similar results were reported in a study of 28 patients with extrahepatic CCA. In this study, *Enterococcus*, *Streptococcus*, *Bacteroides*, *Klebsiella*, and *Pyramidobacter* were most abundant in the bile of patients with cholangiocarcinoma [126]. In addition, analysis of the gut microbiota from stool samples using 16S mRNA in 28 cholangiocarcinoma patients showed that at the genus level, intrahepatic cholangiocarcinoma patients had a higher prevalence of *Lactobacillus*, *Actinomyces*, *Peptostreptococcus*, and *Aloscardovia* compared to cirrhotic and healthy individuals [127]. A limited study of 30 HCC and 35 CCA patients showed that diversity of the basic gut microbiota was associated with better response to anti-PD1 immune checkpoint inhibitor therapy in patients with hepatobiliary pancreatic cancer [128]. Given the interesting data that intratumoral bacteria greatly improve prognosis in pancreatic cancer [129, 130], it is possible that future studies in intrahepatic cholangiocarcinoma will yield similar results. Further research is necessary to investigate the direct causal relationship of these changes in the gut microbiota to disease pathogenesis and the merits of therapeutic intervention.

## Therapeutic applications targeting intestinal bacteria

As previously mentioned, dysbiosis and disruption of the intestinal barrier have been reported in liver diseases, and treatments targeting intestinal bacteria are garnering interest. Clinically, dysbiosis can now be remedied with probiotics, prebiotics, and fecal transplantation (FMT).

Probiotics represent microorganisms that contribute to the suppression of inflammation and the maintenance of homeostasis. They are known to influence host health not only by regulating the balance of intestinal bacteria, but also through mechanisms such as enhancement of the mucosal barrier, antimicrobial action, and immunomodulatory action. Prebiotics are foods that are not degraded or absorbed in the upper part of the digestive tract. Instead, they serve as a selective source of nutrients for beneficial bacteria living in the digestive tract, promoting their growth and improving the intestinal microbiota, thereby contributing to the improvement of human health. Synbiotics are a combination of both probiotics and prebiotics. In clinical practice, probiotics and synbiotics containing *Lactobacillus* and *Bifidobacterium* have been reported to improve the steatosis and liver enzymes in non-alcoholic fatty liver disease (NAFLD) patients [131]. In patients with insulin resistance, the oral intake of *Akkermansia muciniphila* showed weight loss and improvement in insulin sensitivity and liver enzymes [132]. A deficiency of *Lachnospiraceae* and *Ruminococcaceae*, which metabolize carbohydrates and convert them into butyrate, has been observed in patients with alcoholic hepatitis and cirrhosis resulting in decreased blood butyrate levels and contributing to the development of pathological conditions such as hepatitis [133–135]. In fact, evidence suggests that butyric acid from either synbiotics (*Faecalibacterium prausnitzii* and potato starch), tributin, or taken directly, improves intestinal barrier and liver damage [136–138]. Conversely, certain reports have indicated that the effect of probiotics on hepatic encephalopathy was limited [139] and that probiotics for PSC failed to show efficacy [105]. Therefore, further studies on the efficacy of probiotics are warranted. The clinical outcomes of gut bacteria-targeted therapies reported to date for PSC are summarized in Table 1.

FMT is a treatment that is expected to improve dysbiosis by transplanting healthy intestinal bacteria into the gastrointestinal tract of patients and has attracted attention as a treatment for refractory *Clostridioides difficile* infection [140]. In liver diseases, improvement of intestinal permeability, insulin sensitivity, hepatitis, and lipid metabolism were reported in the FMT group in various diseases such as NAFLD [141, 142] and metabolic syndrome [143]. Similarly, improvements in dysbiosis, cognitive function, and hepatitis markers have been reported with FMT in patients with hepatic encephalopathy [144, 145]. A few patients with PSC (10 patients) reported an increase in intestinal bacterial diversity in the FMT group without adverse events, and the effect was sustained for 24 weeks [106] FMT has been reported to improve survival in severe alcoholic hepatitis with a high mortality rate and inadequate effective treatment, and further studies on this are expected [146–148]. Despite this, FMT has been reported to have a 1.4% rate of serious complications related to infection and death [149]. Complications of bacteremia, idiopathic bacterial peritonitis, and fatal cholangitis have been reported during treatment for *Clostridioides difficile*, especially in patients with decompensated cirrhosis [150], suggesting the need for more rigorous donor selection for FMT in the future.

In clinical practice, antimicrobial agents are widely used as treatments that directly target pathogens, but their side effects, such as increased dysbiosis and the emergence of resistant strains associated with long-term use, have become problematic. Recently, bacteriophage therapy, which targets only specific pathogenic bacteria, has been attracting attention again. Bacteriophages minimize changes in the intestinal microbiota, suppress the emergence of resistant bacteria, and are innocuous with minimal impact on the host. In a mouse model of alcohol-related liver disease, hepatitis improved using bacteriophages that targeted *Enterococcus faecalis* [151]. Additionally, the amelioration of liver damage by administration of bacteriophages targeting alcohol-producing *Kp* involved in the pathogenesis of NAFLD [152, 153] has been reported. Bacteriophages targeting *Kp* in IBD patients were also implicated in the reduction of enteritis in a mouse model. More interestingly, these bacteriophages reached the large intestine in combination with esomeprazole when administered to healthy subjects, confirming that they can be used safely without causing changes in the intestinal microbiota [154]. Similarly, our group recently reported the therapeutic potential of targeting specific gut bacteria in PSC using bacteriophages [116]. A comparison of the intestinal microbiota of bacteriophage-treated and non-treated groups after the administration of *Kp* from PSC patients to specific pathogen-free (SPF) mice showed that in the phage-treated group, phage administration reduced the amount of *Kp* and improved DDC-induced hepatobiliary injuries [116]. Bacteriophage therapy against the liver disease model is summarized in Table 2. It is expected that bacteriophage therapy will become a potential option for liver diseases in the future.

**Table 2:** Bacteriophage therapy against liver disease model

Target bacteria	Disease	Design	Route of phage administration	Phage and dosage	Outcome	Reference
*E. faecalis*	Alcohol-associated liver disease	GF mice transplanted with faecal microbiota of patients	Oral	3 or 4 pahage (10^9^ PFU) 1 day before ethanol binge	Phages targeting cytolysin-positive Enterococcus faecalis abolished ethanol-induced liver injury and steatosis.	Duan et al. 2019 [151]
*K. pneumoniae*	NAFLD	GF mice transplanted with faecal microbiota of patients	Oral	2 phages before FMT	FMT with phage pretreatment ameliorated steatopepatitis development.	Yuan et al. 2019 [152]
*K. pneumoniae*	NAFLD	GF mice transplanted with faecal microbiota of patients	Oral	1 phage (10^6^ PFU maximum) once a day for 1, 4 or 7 days	Phages targeting alcohol-producing *K. pneumoniae* attenuated steatopepatitis.	Gan et al. 2023 [153]
*K. pneumoniae*	PSC	GF or SPF mice transplanted with *K. pneumoniae* isolated from PSC patients	Oral or intravenous (IV)	4 phages (10^9^ PFU for oral or 10^8^ PFU for IV) every 3 days for 3 weeks	Both oral and IV administration of phages improved *K. pneumoniae* -induced hepatobiliary injury.	Ichikawa et al. 2023 [116]

*NAFLD* Non-alcohol fatty liver disease, *PSC* Primary sclerosing cholangitis, *GF* Germ free, *SPF* Specific pathogen free, *PFU* Plaque-forming unit, *FMT* Fecal microbiota transplantation

## Conclusion

Intestinal bacteria, along with their metabolites and bile acids, directly influence the immune system and contribute to the pathogenesis of hepatobiliary diseases. The role of the gut microbiota in the pathogenesis of biliary tract cancer has also been elucidated, with detailed mechanisms of both antitumor and tumor-promoting effects. Advances in enterobacterial research in this field are expected to elucidate the detailed pathological mechanism mediated by the gut-liver axis, potentially leading to the clinical application of the gut-liver axis as a diagnostic biomarker and therapeutic target in the future.

## Data Availability

Not applicable.

## Abbreviations

AhRs: Aryl hydrocarbon receptors
ALP: Alkaline phosphatase
AMA: Anti-mitochondrial antibodies
AMPs: Antimicrobial peptides
BT: Bacterial translocation
CCA: Cholangiocarcinoma
CXCR2+: CXC chemokine receptor
DAMPs: Damage-associated molecular patterns
DCs: Dendritic cells
DDC: 5-Diethoxycarbonyl-1,4-dihydrocollidine
DSS: Dextran sodium sulfate
*Eg*: *Enterococcus gallinarum*
FGF: Fibroblast growth factor
FOXP3: Forkhead box P3
FXR: Farnesoid X receptor
GALT: Gut-associated lymphoid tissue
GPCRs: G protein-coupled receptors
GVB: Gut-vascular barrier
HBx: HBV-encoded oncogene X protein
HCC: Hepatocellular carcinoma
IBD: Inflammatory bowel disease
IELs: Intraepithelial lymphocytes
IFN-γ: Interferon-gamma
IgA: Immunoglobulin A
*Kp*: *Klebsiella pneumoniae*
LCA: Lithocholic acid
LPS: Lipopolysaccharides
M cells: Microfold cells
MAIT: Mucosal-associated invariant T cells
MLNs: Mesenteric lymph nodes
MyD88: Myeloid differentiation primary response 88
NASH: Non-alcoholic steatohepatitis
NF-κB: Nuclear factor kappa B
NKT cells: Natural killer T cells
NLRP3: NOD-like receptor protein 3
NLRs: NOD-like receptors
NOD1: Nucleotide-binding oligomerization domain-containing protein 1
PAMPs: Pathogen-associated molecular patterns
PBC: Primary biliary cholangitis
PDC-E2: Pyruvate dehydratase complex E2 component
*Pm*: *Proteus mirabilis*
PRR: Pattern recognition receptor
PSC: Primary sclerosing cholangitis
SBP: Spontaneous bacterial peritonitis
SCFAs: Short-chain fatty acids
SIBO: Small intestinal bacterial overgrowth
TGR5: Takeda-G-protein-receptor-5
TLR2: Toll-like receptor 2
TNF-a: Tumor necrosis factor alpha
UDCA: Ursodeoxycholic acid
CXCL1: CXC motif chemokine ligand 1
FMT: Fecal transplantation
NAFLD: Non-alcoholic fatty liver disease
SPF: Specific pathogen-free
